# LncRNA-1810034E14Rik reduces microglia activation in experimental ischemic stroke

**DOI:** 10.1186/s12974-019-1464-x

**Published:** 2019-04-08

**Authors:** Xi Zhang, Xiao-Lei Zhu, Bi-Ying Ji, Xiang Cao, Lin-Jie Yu, Yan Zhang, Xin-Yu Bao, Yun Xu, Jia-Li Jin

**Affiliations:** 1 Department of Neurology, Drum Tower Hospital, Medical School and The State Key Laboratory of Pharmaceutical Biotechnology, Nanjing University, Nanjing, 210008 China; 2 Jiangsu Key Laboratory for Molecular Medicine, Medical School of Nanjing University, Nanjing, 210008 China; 3 Jiangsu Province Stroke Center for Diagnosis and Therapy, Nanjing, 210008 China; 4 Nanjing Neuropsychiatry Clinic Medical Center, Nanjing, 210008 China

**Keywords:** Ischemic stroke, lncRNA-1810034E14Rik, Microglial cells, p65

## Abstract

**Background:**

Activation of microglial cells plays an important role in neuroinflammation after ischemic stroke. Inhibiting the activation of microglial cells has been suggested as a potential therapeutic approach in the treatment of ischemic stroke.

**Methods:**

Oxygen-glucose deprivation in primary microglial cells and transient middle cerebral artery occlusion (MCAO) in C57BL/6 mice were used as the in vitro and in vivo ischemic stroke models. Microarray analysis was performed to investigate the overall impact of long non-coding RNAs (lncRNAs) on the inflammation status of microglial cells. RT-qPCR was used to evaluate the lncRNA levels and mRNA levels of cytokines and microglial cell markers. ELISA was taken to measure the level of cytokines. Immunofluorescence was used to observe the activation of microglial cells. Western blotting was performed to test the p65 phosphorylation.

**Results:**

In this study, we showed that LncRNA-1810034E14Rik was significantly decreased in LPS-treated or oxygen-glucose deprivation-induced microglial cells. Overexpression of 1810034E14Rik decreased the infarct volume and alleviated brain damage in MCAO mice. 1810034E14Rik overexpression reduced the expression of inflammatory cytokines not only in ischemic stroke mice but also in oxygen-glucose deprivation-induced microglial cells. Moreover, 1810034E14Rik overexpression could suppress the activation of microglial cells and inhibit the phosphorylation of p65.

**Conclusions:**

LncRNA-1810034E14Rik plays an anti-inflammatory role in ischemic stroke and regulates p65 phosphorylation, making it a potential target for stroke treatment.

**Electronic supplementary material:**

The online version of this article (10.1186/s12974-019-1464-x) contains supplementary material, which is available to authorized users.

## Background

Ischemic stroke is the second leading cause of death and disability in adults, resulting in a series of sequences, including cognitive impairment and epilepsy [1]. Although the pathophysiological mechanisms of stroke have been extensively studied, the only effective treatment for stroke is intravascular thrombolysis or thrombectomy [1, 2].

Inflammation after stroke is closely related to the progression and prognosis of stroke [3, 4]. However, there is still a lack of clinical approaches to effectively reverse inflammation induced by ischemia. Therefore, the discovery of new targets is particularly important for the development of ischemic stroke therapies.

Long non-coding RNA (LncRNA) is a set of transcripts that are longer than 200 bp, lacking or without an open reading frame (OFR), and that do not necessarily have polynucleotide tails [5–7]. The number of lncRNAs is much lower than that of coding protein genes (mRNAs). Compared with the coding protein gene, it has higher tissue/organ specificity. LncRNA is classified into antisense lncRNAs, intronic lncRNAs, divergent lncRNAs, intergenic lncRNAs, promoter upstream lncRNAs, promoter-associated lncRNAs and transcription start site-associated lncRNAs according to the relative position on the chromosome [8]. The functions of lncRNAs have not been fully clarified. Some studies have found that lncRNAs affect the transcription of the coding gene promoter and interfere with the expression of downstream genes [9, 10]. Additionally, lncRNAs can interfere with the shearing of mRNA and form different shear forms [11]. There has been a great deal of research on lncRNAs in different diseases, but the function of lncRNAs in ischemic stroke is relatively unknown.

Microglial cells are resident immune cells in the central nervous system and act as a sensor in a normal brain [12]. Previous studies have found that microglial activation is associated with inflammation after stroke. Activated microglia secrete a large number of cytokines, such as IL-1b and TNF-α, and attract peripheral neutrophils, macrophages and T cells to infiltrate around the infarcted cortex [12–14]. NF-κB plays a key role in regulating the immune response to infection. Incorrect regulation of NF-κB has been linked to cancer, inflammatory and autoimmune diseases, and improper immune development [15]. It has been well proven that the NF-κB signaling pathway is active during ischemic stroke. Previous studies found that suppressing the NF-κB signaling pathway protected against ischemic stroke by inhibiting excessive microglial activation and partly promoting neuronal survival [16]. Many researchers have observed an elevation of pro-inflammatory cytokine levels, enhanced nuclear NF-κB transcriptional activity, and increased nuclear translocation of NF-κB in experimental ischemia animals [15]. Interference with NF-κB signaling with p50 knockout mice reduced infarct size and helped functional recovery [17]. It has been reported that in Toll-like receptor 2-activated microglia, the subsequent NF-κB mediated production of TNF-α, IL-10, and NO in a time-dependent manner [18]. These reports suggest that suppression of neuroinflammation and the NF-κB pathway may be a potential target for ischemic stroke.

In this study, we found that lncRNA-1810034E14Rik was reduced during OGD/MCAO injury in microglial cells. We observed that 1810034e14Rik protected against ischemic stroke in MCAO mice and overexpression of 1810034e14Rik in microglia could suppress the activation of microglial cells and the release of pro-inflammatory cytokines, which might be associated with inhibition of the NF-κB pathway. Thus, our study demonstrated that 1810034E14Rik played a vital role in cerebral ischemic damage, suggesting that 1810034E14Rik might be a potential target of stroke treatment.

## Methods

### Animals

Male, 7- to 8-week-old C57BL/6 mice (20–23 g in weight) were purchased from Model Animal Research Center of Nanjing University. The animals were raised in a regulated environment (12 h light/dark cycle) and were supplied with clean water and food. All the experiments involving animals were approved by the Institutional Animal Care and Use Committee of Nanjing University.

### Stereotaxic injection and focal ischemia

Mice were anesthetized with 10% chloral hydrate (3.2 ml/kg) by intraperitoneal injection. A lentiviral vector (1 × 10^9^ TU/ml, 1 μl) overexpressing lncRNA-1810034E14Rik (Lv-1810034E14Rik) or negative control (Lv-control) was injected into the cerebral cortex with a stereotaxic instrument after the mice were anesthetized. Each mouse got three injections in the right cortex (0.3 mm front of the bregma, 0.8 mm behind the bregma, and 1.9 mm after the bregma; 3 mm lateral; and 1.8 mm deep). The injection was completed with a 10 μl microsyringe at the rate of 0.1 μl min^−1^.

Two weeks after injection, the mice were subjected to MCAO to induce focal ischemia as previously described [19]. Briefly, 8-week-old male C57BL/6 mice were anesthetized with 1.5% isoflurane in a 68.5% N_2_O/30% O_2_ mixture, the ischemia was induced by occlusion of the right MCA for 1 h. Rectal temperature of each mouse was maintained at 37 ± 0.5 °C by a heating pad during the surgery.

Male C57BL/6J mice were randomly assigned to 4 groups (*N* = 15): sham, MCAO 60 min/24 h reperfusion, MCAO 60 min/24 h reperfusion + Lv-control (stereotactic injection of negative control lentivirus into cortex 2 weeks before MCAO), MCAO 60 min/24 h reperfusion + Lv-LncRNA1810034E14Rik (stereotactic injection of lentivirus overexpressing 1810034E14Rik into cortex 2 weeks before MCAO).

### Neurological function evaluation

The Rota-rod test, grip strength, and neurological symptom score (Additional file 1) were performed to assess neurological functions as previously described [20]. Investigators were blind to mouse group assignments.

### Cell culture and oxygen-glucose deprivation

Primary microglial cells were prepared from postnatal 1–2 days C57BL/6 mice. After extracting the brain, the meninges were removed and the entire cortex in Leibovitz’s L15 medium was isolated (Gibco Life Technologies). Then the nylon membrane (pore size of 70 mu m cell filter; Falcon, Pittsburgh, PA, USA) was used to dissociate the tissue mechanically. After centrifugation, the precipitates were suspended in Dulbecco’s modified Eagle’s medium, 10% fetal bovine serum (Myoclone; Gibco), and 1% gentamicin (Gibco Life Technologies). Cells were grown in 5% CO2 at 37 °C, and the medium was half-changed at day 5. After culturing for 2 weeks, flasks were shaken gently and supernatant containing microglial cells was collected [21]. The obtained microglia cells were seeded into 24-well plates at a number of 5 × 10^5^ per well for 6 h to adhere.

The mature microglial cells were cultured with Dulbecco’s modified Eagle’s medium, 10% fetal bovine serum (Myoclone; Gibco) and 1% gentamicin (Gibco Life Technologies) and were treated with LPS (100 ng/ml, *Escherichia coli* 055: B5, Sigma, USA) for 3 h or 6 h, and total RNA was extracted using a TRIzol commercial kit (Invitrogen, USA).

After transfection with Lv-1810034E14Rik for 48 h, microglial cells were exposed to OGD for 4 h to imitate ischemia in vitro. Briefly, cultures were changed from the normal medium to the glucose-free medium. After flushing for 15 min with 5%CO2/95%N2 at 2 psi (1 psi = 6.89 kPA), microglial cells were put in a hypoxia chamber (Billups-Rothenberg, Del Mar, CA). Then chambers were placed at 37 °C for 4 h. After OGD is ending, cells were returned to the normal medium and grown at normal conditions for 24 h.

Primary cortical neurons were prepared from E16–17 mouse embryos. Cortices were dissected, treated with trypsin, and plated at 4 × 10^5^ cell/ml on poly-D-lysine-coated 24-well plates or glass coverslips. Cells were grown in Neurobasal media supplemented with estrogen-free B27 supplement (Invitrogen, Carlsbad, CA, USA) and 25 nM glutamine at 37 °C in a humidified 5% CO2 incubator.

### RNA interference

Small interfering RNA (siRNA) targeted at mouse 1810034E14Rik was used to silence 1810034E14Rik. A mixture of siRNA (100 μmol/L) and Lipofectamine RNAiMAX Transfection Reagent (Invitrogen, Carlsbad, CA, USA) was incubated at room temperature for 15 min. The mix was added to microglial cells and incubated for 24 h. The sequence of siRNA is 5′-GCCAGGAGAAACACTTTGA-3′ (forward), 5′-GAGCAGTCCTTCGAATACT-3′ (reverse). The sequence of nonsense control siRNA is 5′-UUC UCC GAA CGU GUC ACG UTT-3′ (forward), 5′-ACG UGA CAC GUU CGG AGA ATT-3′ (reverse).

### Cell viability assessment

After being treated with microglia-free supernatants for 24 h, LDH release assay (Beyotime Biotechnology, China) and cell counting kit-8 (CCK-8) analysis were used to measure the cell viability. In brief, 10 μl of CCK-8 solution (5 mg/ml; Sigma) was added to each well in incubated at 37 °C for 2 h. Then, the absorbance at 450 nm was measured with a microplate reader.

### Microarray analysis

Total RNA of the cells was extracted by using a TRIzol commercial kit (Invitrogen, USA). And Quick Amp Labeling Kit, One-Color (Agilent, USA) was used to prepare labeling reaction. Then labeled/amplified RNA and labeled cRNA QC were purified by RNeasy Mini Kit (Qiagen, German). After fragmentation, hybridization, and microarray wash, the microarray was scanned by Agilent Microarray Scanner (Agilent, USA). LncRNAs with differential expressions in primary microglial cells were picked out by the whole genome microarray expression profiling with the fold change > 2 and adjusted *P* < 0.05. The microarray analysis was performed with Agilent Feature Extraction by Oebiotech, Shanghai, China. In addition, sample preparation and microarray hybridization were performed according to the manufacturer’s standard protocol with only minor modifications.

### Real-time polymerase chain reaction

TRIzol commercial kit (Invitrogen, USA) was used to extract total RNA of the cells and tissues. The cDNA was synthesized using a reverse transcriptase kit (Takara, Japan). The primer sequences are listed in Additional file 2.

### Cytokine and chemokine measurements

The levels of IL-1b, IL-6, TNF-α, IL-4, and IL-10 in the supernatant of cell culture and cortical tissues were detected by the ELISA Kits (R&D Systems, USA). The process was performed according to the instructions.

### Western blotting

Protein from cortex was extracted and then quantified with Thermo Scientific BCA kit (USA). NE-PER Nuclear and Cytoplasmic Extraction Reagents was used for nucleo-cytoplasmic separation, and the operation was performed according to the protocol. In brief, 100 μl Reagent A was added to the microglial cells. Twenty minutes later, centrifuging for 10 min at 3000×*g* and 4 °C, collecting the supernatant. 150 μl Reagent B was added to the sediment. Twenty minutes later, centrifuging for 10 min at 12000 g and 4 °C, collecting the supernatant. Equal amounts of protein samples were separated by SDS-PAGE and blotted onto polyvinylidene fluoride (PVDF) membranes. The membranes were probed with primary antibodies against CD16 (ab 203,883, 1:1000), CD11b (ab13357, 1:1000), p-IKK (CST2697, 1:1000), p-p65(CST3033, 1:1000), p65(CST8242, 1:1000), IκB (CST4812, 1:1000). GAPDH (CST5174, 1:5000) was used as a loading control. The secondary antibodies were goat anti-rabbit or anti-mouse IgG (H + L) HRP (Bioworld Technology, USA) and exposed to film.

### Immunofluorescence

Cells/brain slices (20um) were fixed with 4% formalin, washed with PBS for three times, and then blocked by 2% BSA for 2 h at room temperature. The samples were incubated with following primary antibodies at 4 °C overnight: antibodies for Iba1 (ab48004, 1: 200), NeuN (ab104224, 1:200), Cleaved-caspase3 (CST9664, 1:200), TMEM119 (ab209064, 1:200). Primary antibodies were detected by using goat anti-rabbit or anti-mouse secondary antibodies (1:200, Invitrogen, USA) for 1.5 h at room temperature. After washed with PBS for three times, the cells/brain slices were stained with 100 nM DAPI (Sigma, USA) for 15 min. Samples were observed and photographed (AX10, ZEISS, Germany).

### Statistical analysis

Experimental results were shown as mean ± SEM, and data were handled with SPSS 15.0 software. For those analyses with only one factor involved, the one-way ANOVA and multiple comparisons followed by Bonferroni tests were applied, while the two-way ANOVA and multiple comparisons followed by Tukey were used when two factors get involved. All statistical tests were two-sided with *P* < 0.05 considered statistically significant.

## Results

### Genome-wide lncRNA and mRNA expression during LPS challenge in microglial cells

**Fig. 1:**
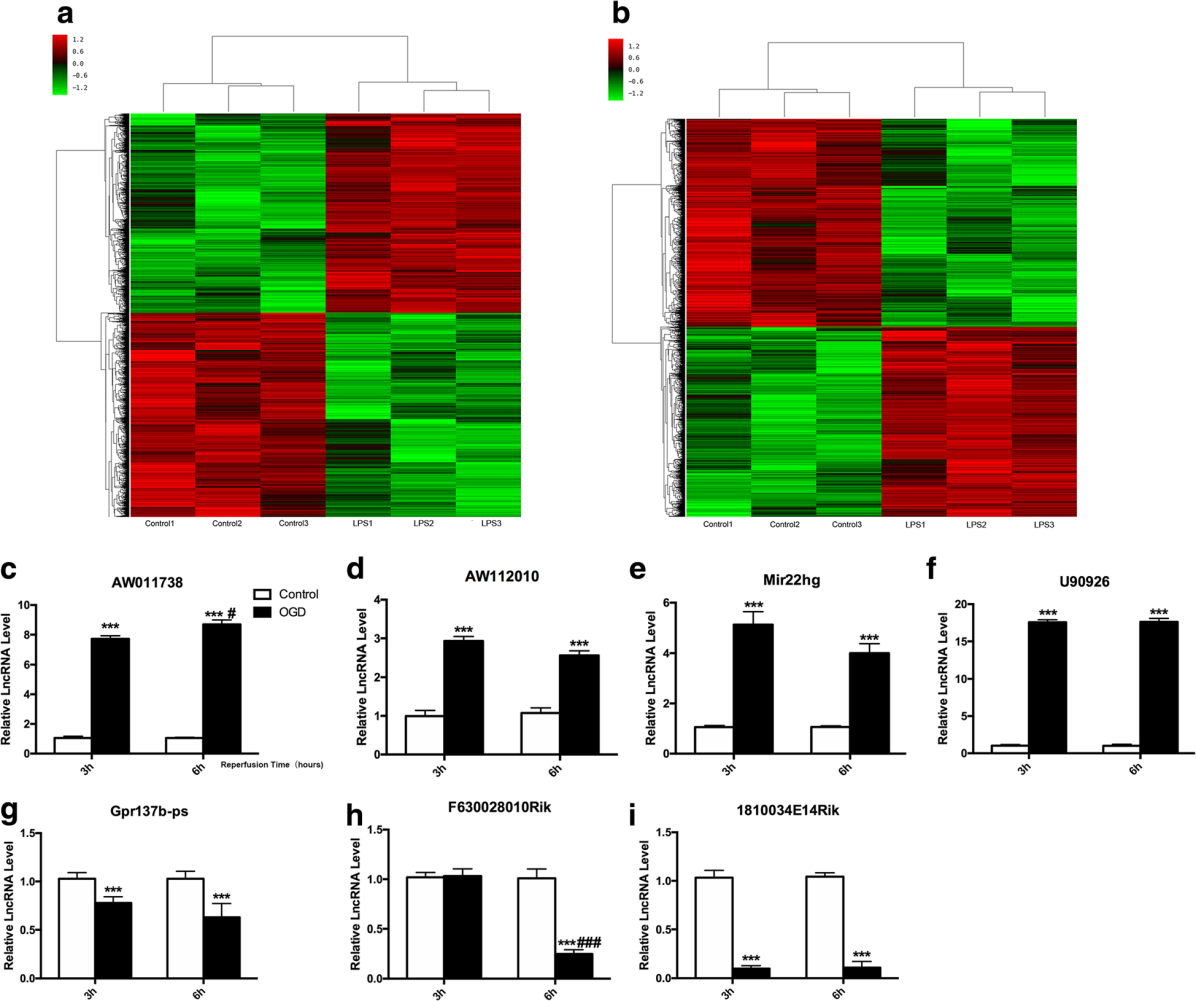
LncRNA expression profile in LPS induced primary microglial cells. **a**, **b** Heat map of lncRNAs in microglial cells stimulated with LPS (100 ng/ml) for 3 h (**a**) or 6 h (**b**). **c–i** RT-qPCR validation of the expression of 7 lncRNAs (AW011738, AW112010, Mir22hg, U90926, Gpr137b-ps, F630028010Rik, and 1810034E14Rik) selected from differentially expressed lncRNAs in microglial cell underwent 4 h OGD/3 h reperfusion or 4 h OGD/6 h reperfusion. The data represents mean ± SEM. *n* = 15, ***P < 0.001 versus the control group;^#^*P* < 0.05, ^###^*P* < 0.001 versus the 4 h OGD/3 h reperfusion group

To determine the overall impact of lncRNAs on the inflammation status of microglial cells, a microarray was performed to analyze the expression of lncRNAs in LPS-induced microglial cells (100 ng/ml). The lncRNA expression profiles showed that 2453 lncRNAs (1337 upregulated/1116 downregulated) and 2367 lncRNAs (1352 upregulated/1015 downregulated) were significantly differentially expressed after 3 and 6 h of LPS stimulation (> 2-fold change, *P* < 0.05). Hierarchical clustering revealed systematic changes in lncRNA expression between control and LPS-induced samples (Fig. 1a, b). Then, we randomly selected seven lncRNAs (AW112010, AW011738, Mir22hg, U90926, Gpr137b-ps, F630028010Rik, and 1810034E14Rik), which showed a greater than 5-fold change, and the RT-qPCR results were consistent with the microarray analysis (Additional file 3). We performed GO and KEGG analysis for each lncRNA co-expressed mRNAs, and according to *P* value, the frequency of each function prediction term was counted, and GO (or KEGG) term with more functional annotations was counted to reflect the overall situation of the functional distribution of lncRNAs. We selected the top 20 lncRNAs (or RNAs) to drawbar graph by frequency. The bioinformatics analysis showed that these statistically significant lncRNAs were correlated with several signaling pathways, including the TNF signaling pathway and Toll-like receptor signaling pathway (Additional file 3). Moreover, these lncRNAs were involved in the innate immune response, immune system process and apoptotic process (Additional file 3), which indicated that these lncRNAs were highly correlated with the inflammation state of microglial cells [15].

From the mRNA expression profiles, 1450 mRNAs (668 upregulated and 782 downregulated) were significantly differentially expressed between the control and the LPS-induced samples (≥ 2-fold change, *P* ≤ 0.05) 3 h or 6 h. Thirty-three mRNAs were upregulated nearly 100-fold, and these mRNAs are mainly involved in the immune response. We observed that mRNAs involved in the NF-κB signaling pathway were extremely upregulated after LPS induction, which mean the NF-κB signaling pathway was activated.

### LncRNA-1810034E14Rik was significantly decreased in microglial cells after OGD

To explore the role of specific lncRNAs involved in ischemia and hypoxia, we examined the mRNA levels of 7 lncRNAs (AW112010, AW011738, Mir22hg, U90926, Gpr137b-ps, F630028010Rik, and 1810034E14Rik) in microglial cells exposed to 4 h OGD with 3 h or 6 h reperfusion. The results showed that AW112010, AW011738, Mir22hg, and U90926 were significantly increased in 4 h OGD-treated microglial cells with 3 h and 6 h reperfusion (Fig. 1a–f), while Gpr137b-ps, F630028010Rik, and 1810034E14Rik were significantly reduced (Fig. 1g–i). Notably, lncRNA-1810034E14Rik was highly expressed in microglial cells and decreased significantly after OGD treatment. And we also tested the change of 1810034E14Rik in neurons, astrocytes, and endothelial cells after OGD induce, but no significant change was observed (Additional file 4).

### Upregulation of 1810034E14Rik affected the biological function of microglial cells

**Fig. 2:**
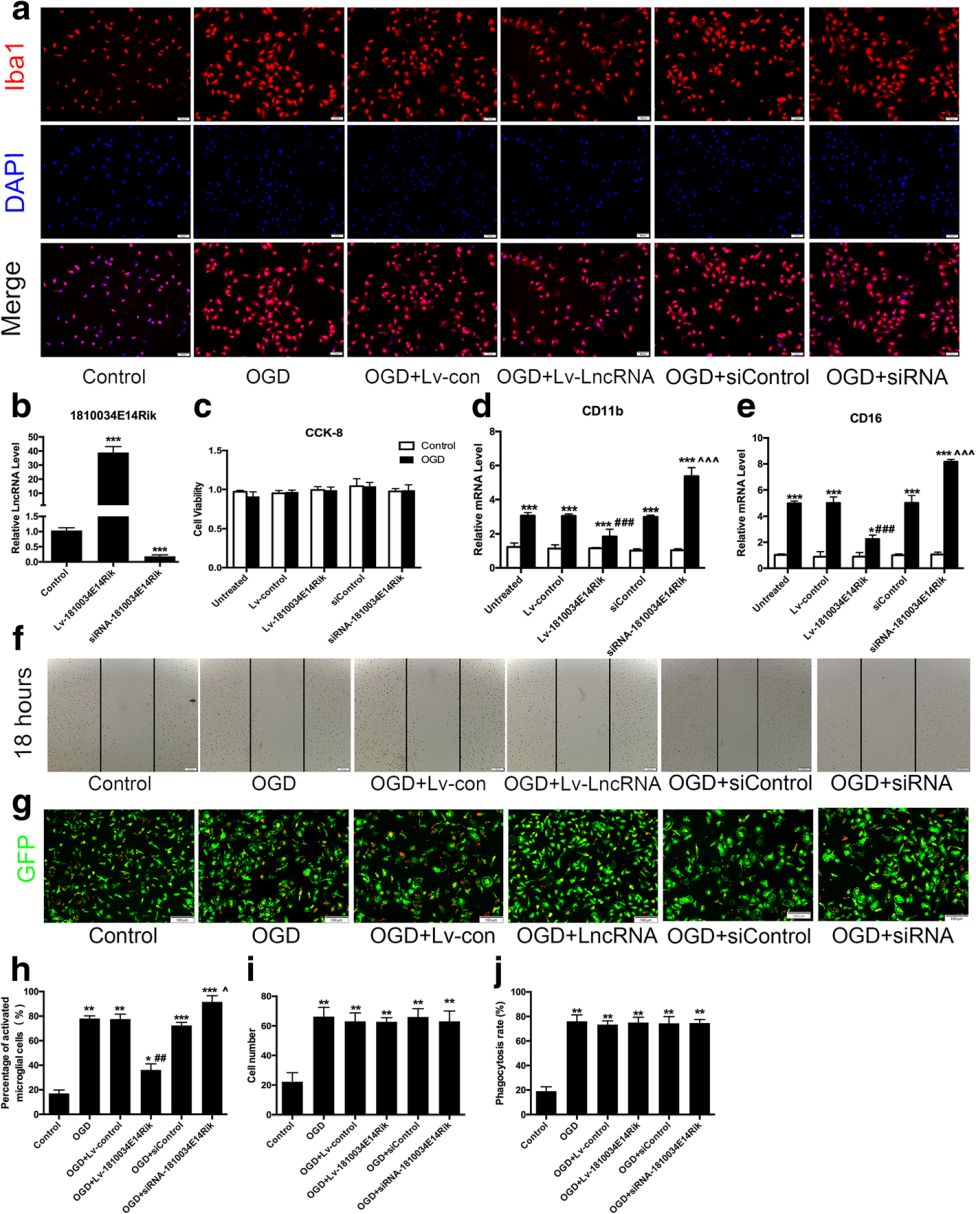
Effects of 1810034E14Rik on the biological function of microglial cells. **a** Primary microglia were transfected with 1810034E14Rik or siRNA-1810034E14Rik for 24 h and then challenged with 4 h OGD/24 h reperfusion to induce an ischemic state. Immunofluorescence staining of Iba1. **b** RT-qPCR validation of the expression of lncRNA-1810034E14Rik. **c** CCK-8 analysis of the viability of microglial cells. **d**, **e** RT-qPCR validation of the expression of CD11b **d** and CD16 **e**. **f** Cell migration of microglia was measured by scratch assay, and wound closure at 18 h was used to compare cell motility. **g** 5 μm red fluorescent microspheres (10^6^/ml) were added to culture to observe the phagocytosis of the microglia. **h** Quantification of Fig. 2a. **i** Quantification of Fig. 2f. **j** Quantification of Fig. 2g. The data represents mean ± SEM. *n* = 15, *P < 0.05, **P < 0.01, and ***P < 0.001 versus the control group; ##P < 0.01, ###P < 0.001 versus the OGD + Lv-control group; ^*P* < 0.05, ^^^*P* < 0.001 versus the OGD + siControl group

It has been reported that the main biological functions of microglial cells include inflammation, migration, phagocytosis, and chemotaxis. To investigate the effects of lncRNA-1810034E14Rik on microglial functions, a lentivirus overexpressing lncRNA-1810034E14Rik or a negative control was used to infect primary microglial cells (MOI = 20). As shown in Fig. 2a, h, the microglia change to a more activate morphology with a flattened cytoplasm in culture after 4 h of OGD/24 h of reperfusion. However, overexpression of lncRNA-1810034E14Rik reduced activated microglial cells, while knock-down of 1810034E14Rik increased activated microglial cells. And CCK-8 analysis showed that OGD had little effect on the viability of microglial cells (Fig. 2c). We also tested markers of pro-inflammatory microglial cells by RT-qPCR. OGD-induced upregulation of CD11b and CD16 was partially reversed by 1810034E14Rik overexpression and worsen by knock-down (Fig. 2d, e), which indicated that 1810034E14Rik reduced microglial activation induced by OGD. Subsequently, we detected the migration of microglial cells by scratch tests. The migration ability of microglia was increased after OGD, while 1810034E14Rik have no effect on the migration of microglia (Fig. 2f, i). To examine the phagocytic ability of microglial cells, 5 μm red fluorescence microspheres were added to the culture of microglia after OGD (10^7^/ml), and the phagocytosis of the microglia was observed after 3 h under a fluorescence microscope. The results showed that the number of microglia cells phagocytizing fluorescence microspheres increased after OGD, but 1810034E14Rik had no significant effect on the phagocytic function of microglial cells (Fig. 2g, j).

### Overexpression of 1810034E14Rik alleviated the inflammation status of microglial cells

**Fig. 3:**
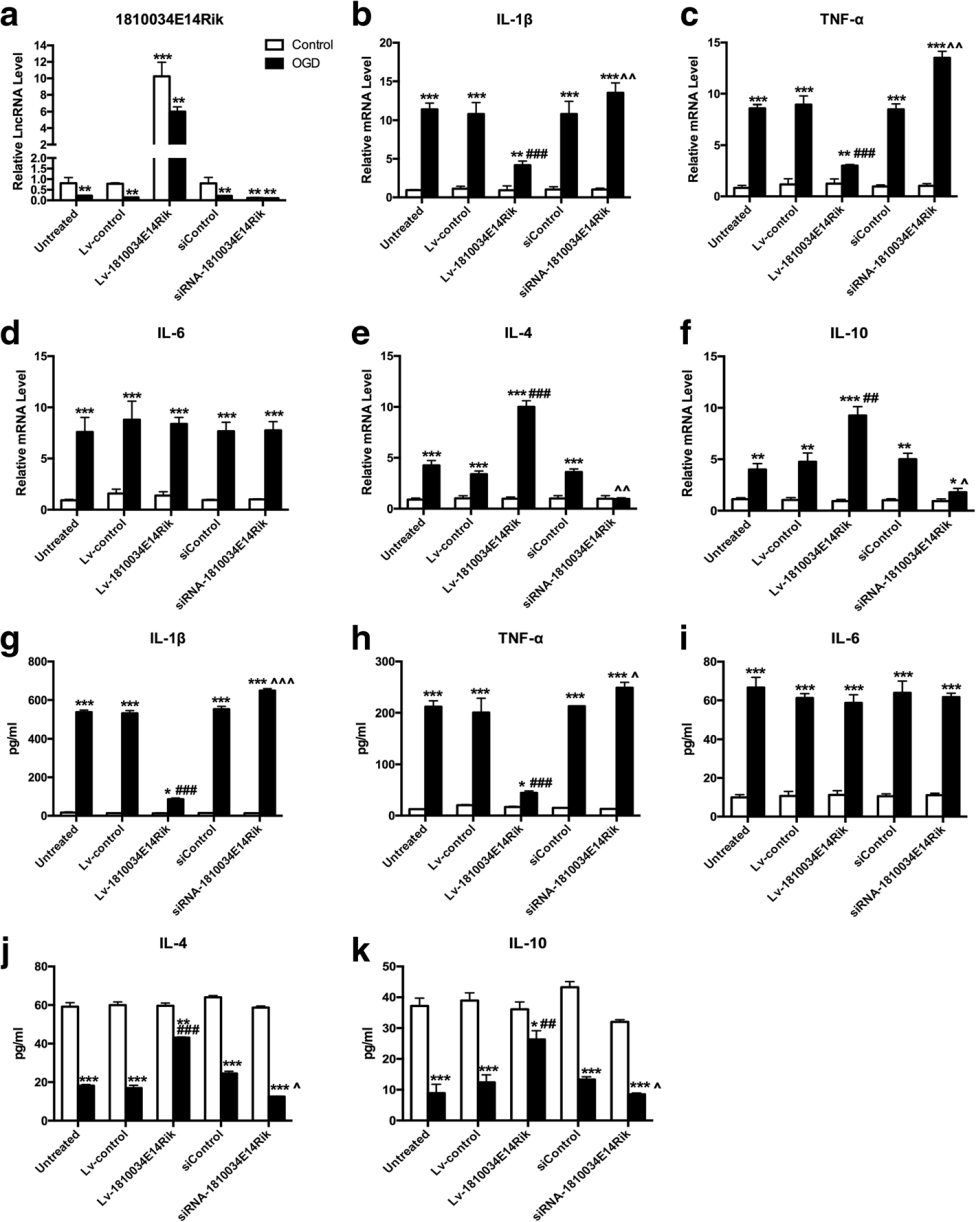
Overexpression of 1810034E14Rik downregulated inflammatory factors in microglial cells. **a** The primary microglial cells were transferred with Lv-1810034E14Rik or negative control 24 h before OGD 4 h/R 24 h induce. The expression of lncRNA-1810034E14Rik was detected by RT-qPCR. The mRNA levels of cytokines IL-1b (**b**), TNF-α **c**, IL-6 (**d**), IL-10 (**e**), and IL-4 (**f**) in primary microglial cells were determined by RT-qPCR. Cytokines such as TNF-α (**g**), IL-1b (**h**), IL-6 (**i**), IL-4 (**j**), and IL10 (**k**) secreted by microglial cells were tested by ELISA. The data represents mean ± SEM. *n* = 15, *P < 0.05, **P < 0.01, and ***P < 0.001 versus the control group; ^##^*P* < 0.01, ^###^*P* < 0.001 versus the OGD group + Lv-control group; ^*P* < 0.05, ^^*P* < 0.01, ^^^*P* < 0.001 versus the OGD + siControl group

It is well known that inflammation induced by microglial cells is closely related to their activation. To observe the difference in microglial inflammation induced by OGD, we examined the changes in inflammatory factors in microglial cells by RT-qPCR. The inflammatory factors TNF-α, IL-6, and IL-1b were significantly increased after 4 h of OGD/24 h of reperfusion, and overexpression of lncRNA-1810034E14Rik reduced TNF-α and IL-1b but not IL-6. The levels of IL-4 and IL-10 were elevated after OGD and significantly increased after overexpression of lncRNA-1810034E14Rik, while knockdown of 1810034E14Rik aggravated inflammation status of microglial cells (Fig. 3b–f). Moreover, the inflammatory factors secreted by microglial cells in culture were detected by ELISA. Overexpression of lncRNA-1810034E14Rik significantly reduced TNF-α and IL-1b and increased IL-4 and IL-10 in OGD-induced microglial cells (Fig. 3g–k), which was consistent with the results of RT-qPCR. Therefore, LncRNA-1810034E14Rik could suppress the inflammation status of microglial cells caused by OGD.

### 1810034E14Rik attenuated neuronal damage induced by microglial cells

**Fig. 4:**
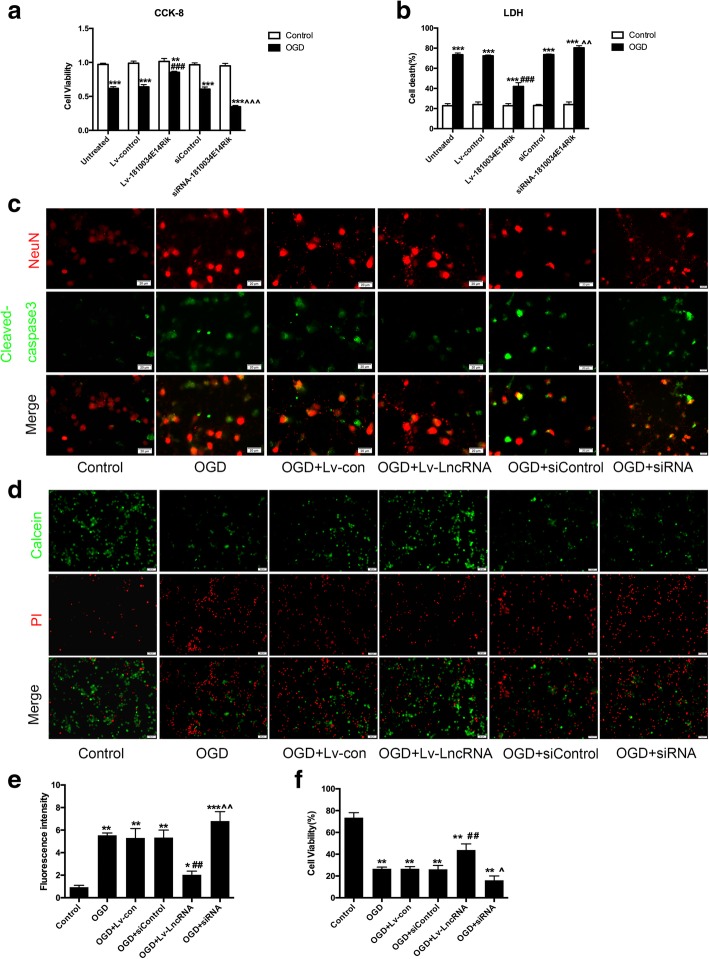
1810034E14Rik ameliorated microglial cells induced neuron injury. **a** Supernatant culture of microglial cells with different treatment was collected and added to 5-day-old neurons at 1:4. Neuron death was measured by LDH released assay. **b** Cell counting Kit-8 assay of neurons treated with the culture of microglial cells. **c** Representative double-staining immunofluorescence of NeuN and cleaved-caspase3 in neurons. **d** Calcein-AM/PI co-staining immunofluorescence of Neurons. The data represents mean ± SEM. *n* = 15, *P < 0.05, **P < 0.01, and ***P < 0.001 versus the control group; ^##^*P* < 0.01, ^###^*P* < 0.001 versus the OGD group + Lv-control group; ^*P* < 0.05, ^^*P* < 0.01, ^^^*P* < 0.001 versus the OGD + siControl group

To elucidate the effects of microglial cells on neurons, we established neuronal microglial co-cultures. We used a conditioned medium transfer system without cell–cell contact. After transfection with a lentiviral vector that overexpressed lncRNA-1810034E14Rik for 24 h (MOI = 20), primary microglial cells underwent 4 h OGD/24 h reperfusion. Then, microglia-free supernatants were collected and added to 5-day-old neurons. Neuronal survival was quantified 24 h later. LDH release assay and cell counting kit-8 analysis showed that the death of neurons was increased and cell viability was decreased after treatment with supernatants of OGD-induced microglial cells. However, supernatants of OGD-induced microglial cells transfected with lncRNA-1810034E14Rik showed less cytotoxicity to neurons (Fig. 4a, b). Similar results also appeared in cleaved-caspase3 immunofluorescence. Cleaved-caspase3 was observed around the nucleus of neurons that were treated with supernatants of OGD-induced microglial cells. Additionally, there was less cleaved caspase-3 around the nucleus of neurons in the OGD + lncRAN-1810034E14Rik group (Fig. 4c). Calcein-AM/PI Double staining also confirmed that overexpression of lncRNA-1810034E14Rik could mitigate neuron damage caused by OGD-induced microglial cells, and knockdown of 1810034E14Rik had the opposite effects (Fig. 4d).

### 1810034E14Rik ameliorated ischemic brain injury in vivo

**Fig. 5:**
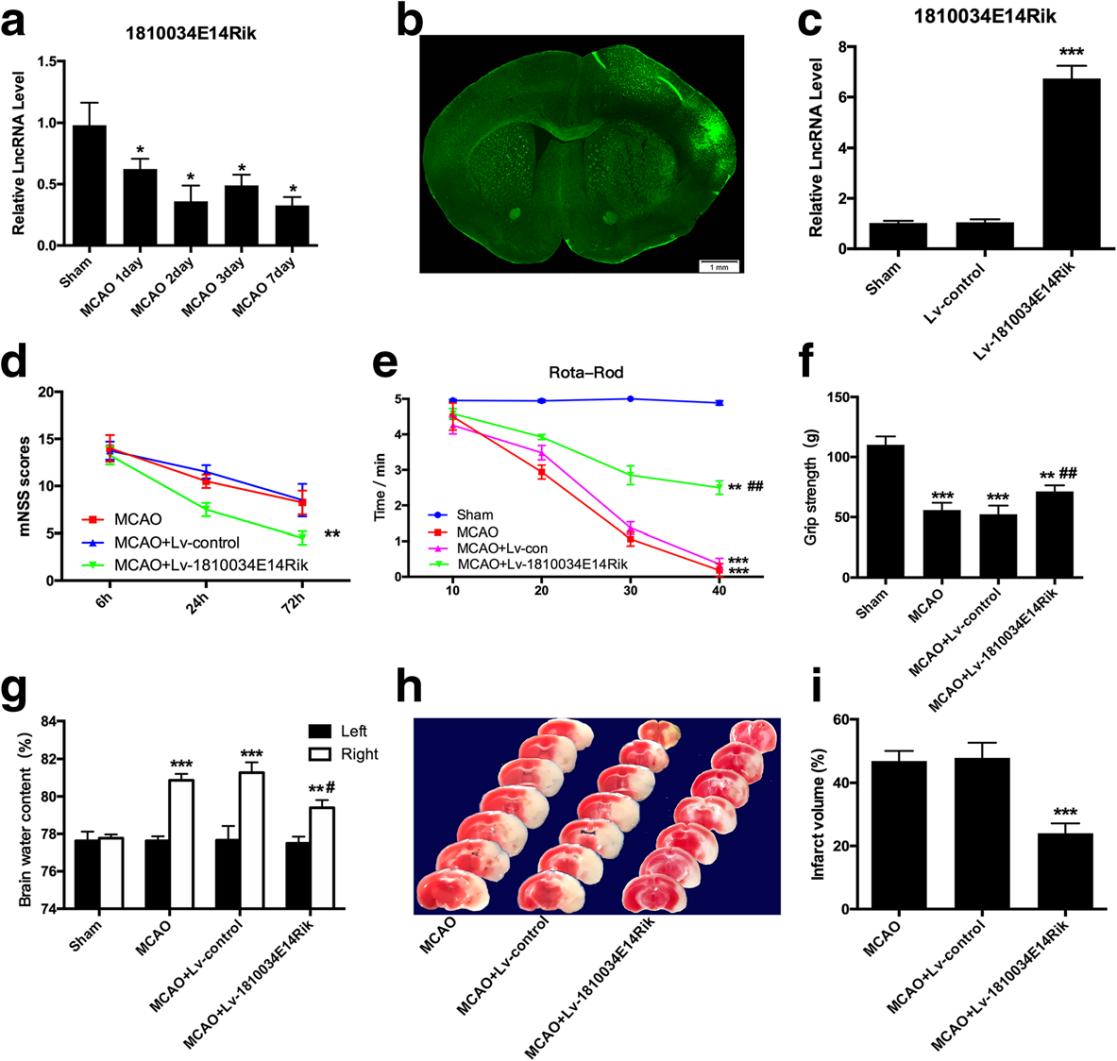
1810034E14Rik improved brain damage after 1 h MCAO/24 h reperfusion. **a** The expression of lncRNA-1810034E14Rik in the infarcted cortex at day 1, day 2, day 3, and day 7 was tested by RT-qPCR. **b** A 1810034E14Rik lentiviral vector expressing GFP was infected in the mice cortex by a stereotactic instrument. **c** The expression of 1810034E14Rik was examined by RT-qPCR in the cortex after injection. The neurological severity scores (**d**), rota-rod test (**e**), and grip strength (**f**) were performed to evaluate functional outcome after MCAO. **g** Brain water content was examined to measure brain edema severity. **h**, **i** Infarct volume after MCAO was confirmed by TTC staining. The data represents mean ± SEM. *n* = 15, *P < 0.05, **P < 0.01, and ***P < 0.001 versus the sham group; ^#^*P* < 0.05 and ^##^*P* < 0.01 versus the MCAO group

To investigate the role of lncRNA-1810034E14Rik in ischemic stroke in vivo, we first detected its level in the cortex of MCAO mice. As shown in Fig. 5a, lncRNA-1810034E14Rik was significantly downregulated in a time-dependent manner. And then we tested the change of other 5 lncRNAs(U90926, Gpr137b-ps, F630028010Rik, Mir22hg and AW0011738) in cortex of MCAO mice (Additional file 5). We observed that 1810034E14Rik had no effects on cerebral reperfusion after MCAO (Additional file 6). In addition, the relative level of lncRNA-1810034E14Rik was significantly increased after Lv-1810034E14Rik injection (Fig. 5b, c). Then, we tested the motor function of mice after MCAO. The results showed that the exercise performance of mice was seriously impaired, but the MCAO+Lv-lnc1810034E14Rik group showed less motor function damage compared to the MCAO + Lv-control group (Fig. 5d–f described separately). In addition, lncRNA-1810034E14Rik alleviated the water content of the infarcted cortex after MCAO (Fig. 5g). After reperfusion, 1810034E14Rik-overexpressing mice had smaller infarcts by TTC staining (Fig. 5h, i). These results suggest that overexpression of 1810034E14Rik attenuated ischemic brain damage.

### Overexpression of 1810034E14Rik decreased pro-inflammatory cytokine levels in vivo

**Fig. 6:**
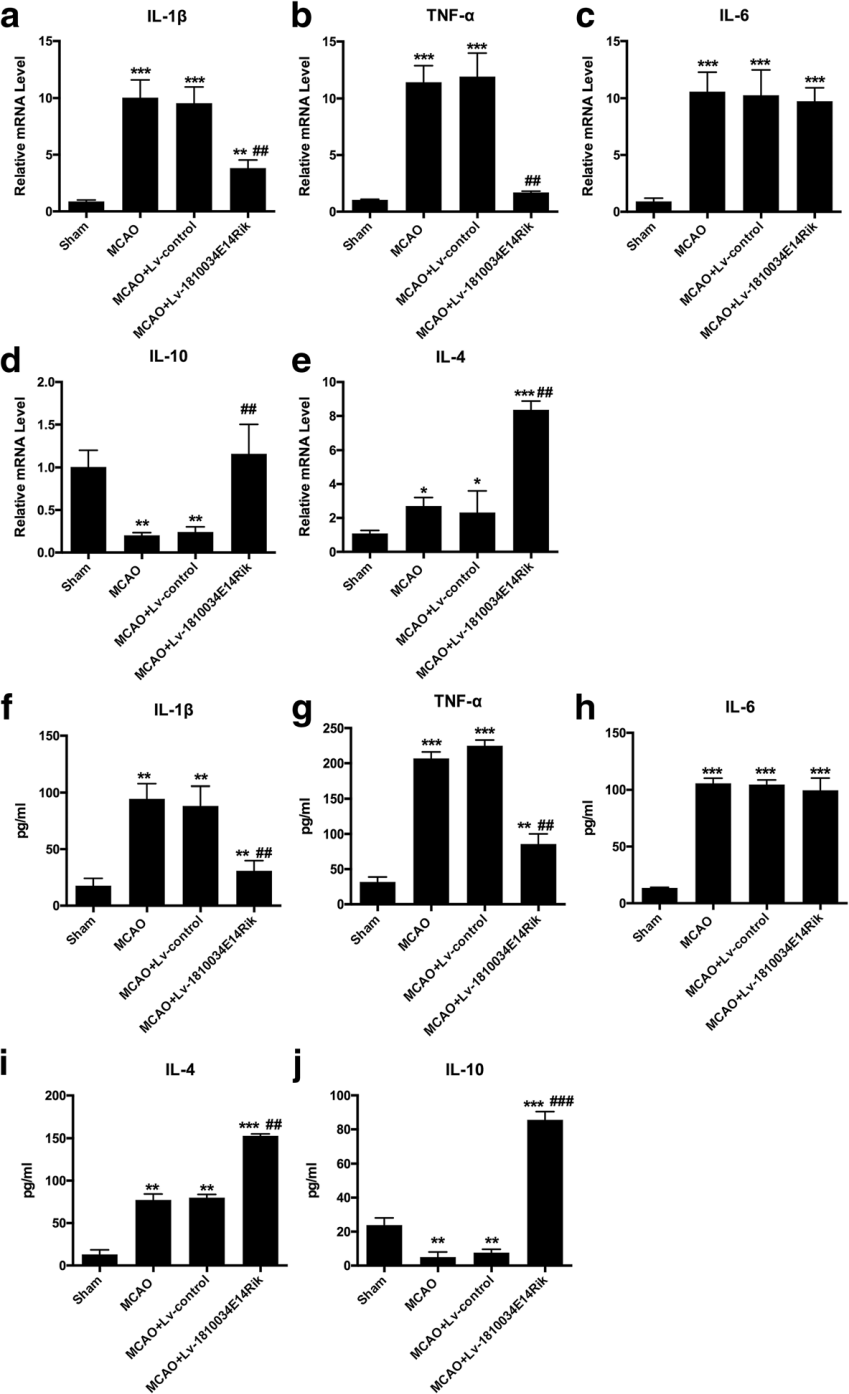
Effects of 1810034E14Rik on inflammatory cytokines after MCAO. The mRNA levels of IL-1b (**a**), TNF-α (**b**), IL-6 (**c**), IL-10 (**d**), and IL-4 **e** in the infarcted cortex at 24 h after MCAO were detected by RT-qPCR. The protein levels of IL-1b (**f**), TNF-α (**g**), IL-6 (**h**), IL-4 (**i**), and IL-10 (**j**) in the infarcted cortex at 24 h after MCAO were examined by ELISA. The data represents mean ± SEM. *n* = 15, **P < 0.01, and ***P < 0.001 versus the sham group; ^#^*P* < 0.05, ^##^*P* < 0.01, and ^###^*P* < 0.001 versus the MCAO group

It has been widely accepted that inflammation contributes to ischemic brain injury. We therefore detected whether altered expression of 1810034E14Rik could regulate the inflammatory response. Using RT-qPCR, we measured the mRNA levels of three pro-inflammatory cytokines, IL-1b (Fig. 6a), TNF-α (Fig. 6b), and IL-6 (Fig. 6c), and two anti-inflammatory cytokines, IL-10 (Fig. 6d) and IL-4 (Fig. 6e), in the infarcted cortex of mice. MCAO significantly increased the mRNA production of all three pro-inflammatory factors. Overexpression of 1810034E14Rik reduced MCAO-induced IL-1b and TNF-α levels and enhanced IL-10 and IL-4 levels. The results of the ELISA assay supported the mRNA change in the infarcted cortex (Fig. 6f–j). These data suggested that 1810034E14Rik has anti-inflammatory roles in the ischemic stroke model.

### Overexpression of 1810034E14Rik reduced the activation of microglial cells in vivo

**Fig. 7:**
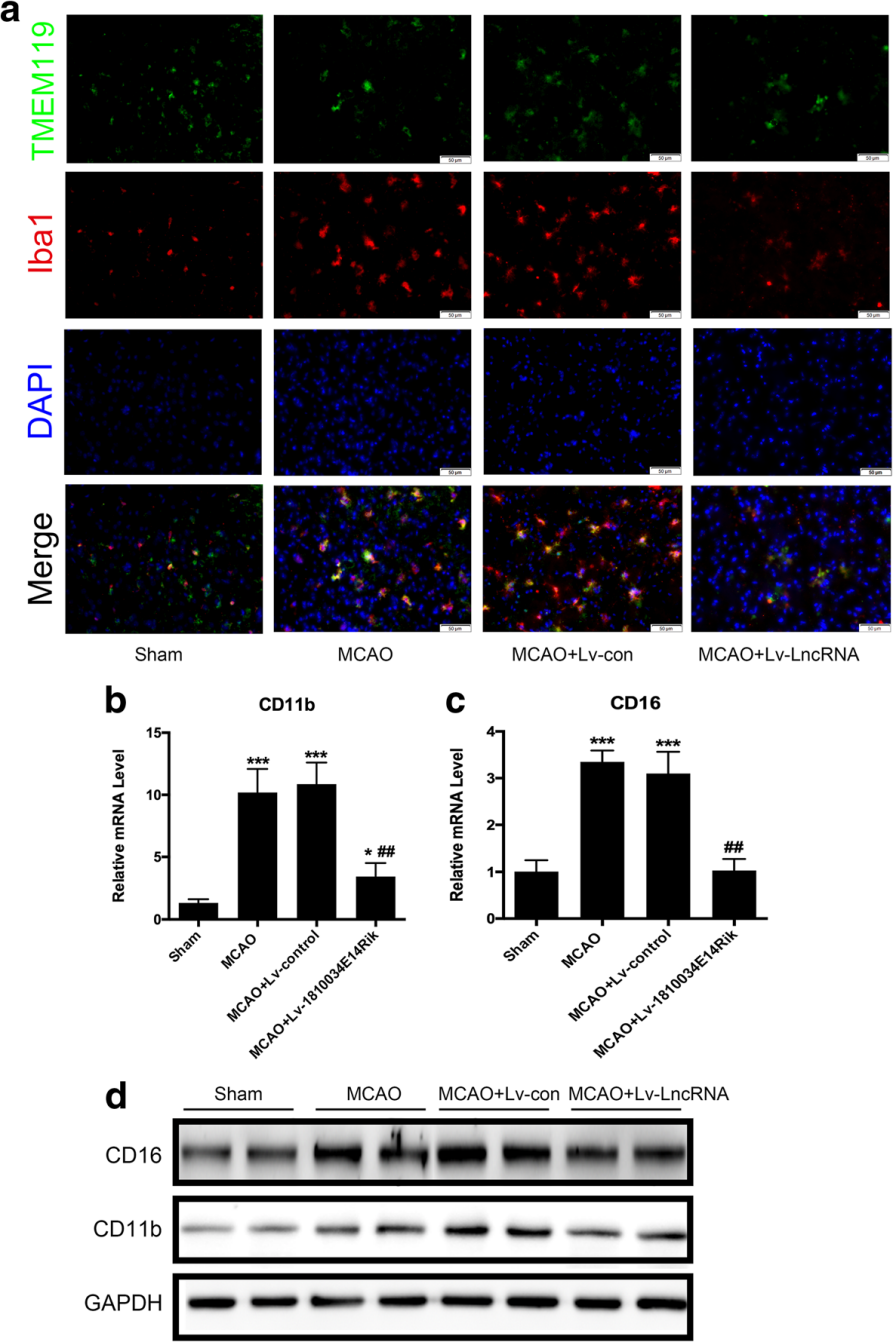
1810034E14Rik overexpression reversed MCAO induced microglia activation. **a** Representative staining immunofluorescence of Iba1+ and TMEM119+ cells. RT-qPCR for CD11b **b** and CD16 **c** in the infarcted cortex. **d** Western blotting of CD11b and CD16 expression in whole ipsilateral cortex tissue. The data represents mean ± SEM. *n* = 15, *P < 0.05 and ***P < 0.001 versus the sham group; ^##^*P* < 0.01 versus the MCAO group

It has been reported that activation of microglial cells is closely related to ischemic brain injury. As shown in Fig. 7a, microglial cells were activated in the ischemic penumbra, and 1810034E14Rik significantly decreased the activation of microglia. Meanwhile, the mRNA and protein levels of two markers of activated microglial cells, CD16 and CD11b, were significantly decreased in MCAO-treated mice after overexpression of 1810034E14Rik (Fig. 7b–d). These results indicated that 1810034E14Rik overexpression ameliorated the activation of microglial cells and the inflammatory response after MCAO treatment.

### 1810034E14Rik reduced activation of microglia probably by inhibiting the NF-κB pathway

**Fig. 8:**
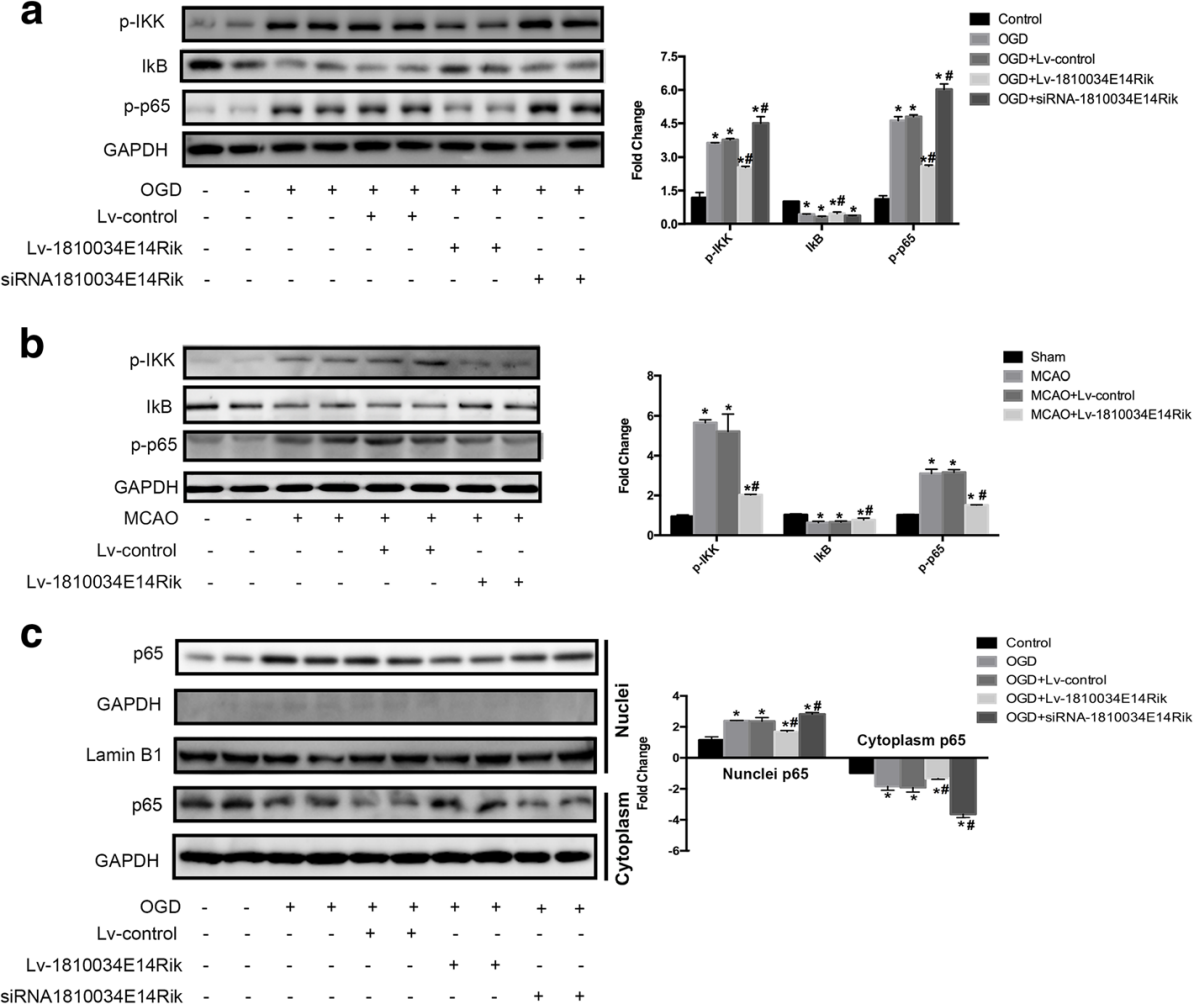
Overexpression of 1810034E14Rik could suppress the activation of NF-κB signaling pathway. **a** Protein levels of p-IKK, IκB, and p-p65 in primary microglial cells were tested by western blotting. **b** Protein levels of p-IKK, IκB, and p-p65 in the infarcted cortex of MCAO mice were examined by western blotting. **c** Protein levels of p65 in nuclei and cytoplasm of primary microglial cells were measured by western blotting separately. The data represents mean ± SEM. *n* = 15, *P < 0.05 versus the sham group or control group; ^#^*P* < 0.05 versus the MCAO group or OGD group

The NF-κB pathway is a classic inflammatory signaling pathway. Our previous research found that the NF-κB pathway in microglial cells was activated after OGD. The MCAO and OGD treatment reduced the level of IκB and the hyperphosphorylation of p65 (Fig. 8a, b). However, overexpression of 1810034E14Rik could suppress the over-activation of the NF-κB pathway. The results of nucleocytoplasmic separation showed that 1810034E14Rik reduced the transfer of p65 to the nucleus after OGD in microglial cells (Fig. 8c). Thus, we conjectured that 1810034E14Rik played an anti-inflammatory role probably by regulating the NF-κB pathway.

## Discussion

It has been widely reported that lncRNAs play an important role in physiological and pathological responses in several diseases, but their function in cerebral ischemia remains unknown [22, 23]. In this study, we found that 1810034E14Rik, a lncRNA associated with inflammatory responses, is downregulated both in OGD- and LPS-induced microglial cells. Using an MCAO mouse model, we found that overexpression of 1810034E14Rik significantly improved behaviors and decreased infarct volume and brain edema in the acute stage. In the infarcted cortex, the pro-inflammatory factors TNF-α and IL-1b were negatively influenced, while the anti-inflammatory factors IL-10 and IL-4 were significantly increased after 1810034E14Rik overexpression. In addition, we determined that the overexpression of 1810034E14Rik could reduce the activation of microglial cells in the infarcted cortex. Taken together, these data suggest a potential role for 1810034E14Rik in regulating the inflammatory response after stroke. Notably, upregulation of 1810034E14Rik suppressed the activation of microglial cells induced by OGD in vitro and affected their biological function, including reduced inflammation. Overall, our studies support that 1810034E14Rik has an improvement in ischemic stroke, and its anti-inflammatory effects are achieved by affecting microglial activation.

Microglial cells, a member of the mononuclear phagocytic cell family, are distributed throughout the central system and are the smallest glial cells, accounting for approximately 5–10% of the total glial cells [24]. As immune effector cells resident in the central nervous system, microglial cells and their mediated neuro-inflammation play a very important role in the damage of the central nervous system and the process of disease progression [25]. Microglial cells have been widely recognized as the primary immune effector in the central nervous system and are involved in many neurological diseases, including ischemic stroke, HIV encephalopathy, Parkinson’s disease, and Alzheimer’s disease [26]. Microglial cells can be activated under pathological conditions, even under very weak stimuli, which manifest as local proliferation and aggregation to different degrees, often accompanied by cell morphology, immunophenotype, and functional changes [27]. Numerous studies have investigated the role of microglial cells in ischemic stroke. Wan S et al. found that polarized microglia occurred dynamically after ischemic stroke, and PAR-1 participated in the activation and polarization of microglial cells [28]. Liesz A et al. reported that the absence of Treg cells augmented the post-ischemic activation of resident and invading inflammatory cells, including microglia and T cells, the main sources of deleterious cerebral tumor necrosis factor-alpha (TNF-α) and interferon-gamma (IFN-gamma), respectively [29]. Li T et al. discovered that CX3CR1(GFP/+) infiltrating cells and reactive microglia represented two distinct populations of cells with different functions and therapeutic potentials for the treatment of stroke [30]. Ma Y et al. summarized the effects of microglia activation on neuronal apoptosis, neurogenesis, and brain function recovery after cerebral ischemia. Differential polarization of microglia may help explain the biphasic effect of microglia on ischemia [31]. In our study, microglial cells were activated in the infarcted cortex after MCAO and secreted IL1, TNF-α, and IL6. However, overexpression of 1810034E14Rik reversed the increase in cytokines, which was consistent with the results in primary microglial cells challenged with OGD.

Inflammation plays a key role in all stages of ischemic stroke. A variety of inflammatory cells and cytokines are involved in this process, a role for lncRNAs in stroke is now being investigated. However, there are many studies on lncRNAs related to their function during stroke. Mehta SL et al. identified that lncRNA-FosDT could modulate post-stroke behavioral deficits and brain damage by its interactions with Sin3a and coREST and subsequent depression of GRIA2, NF-kB, and GRIN1 [32]. Q Xu et al. discovered that lncRNA C2dat1 modulated the expression of CaMKIIδ to impact neuronal survival through the NF-κB signaling pathway and might be a potential target for therapeutic intervention of ischemic brain injury [33]. XJ Zhang et al. found that lncRNA Malat1 could regulate cerebrovascular pathologies in ischemic stroke [34]. Jue Wang et al. reported that H19 promoted neuroinflammation by driving HDAC1-dependent M1 microglial polarization [35]. Wen Y et al. found that lncRNA Gm4419 contributes to OGD/R injury of cerebral microglial cells via IκB phosphorylation and NF-κB activation [36].

1810034E14Rik, a long non-coding RNA, was screened by lncRNA microarray analysis. It was significantly decreased both in OGD-induced microglial cells and in the infarcted cortex. To date, there are no studies on the function of lncRNA-1810034E14Rik in any disease. Bioinformatics suggested that it might be involved in the innate immune response and apoptotic process. Additionally, we validated that 1810034E14Rik could attenuate the inflammatory response caused by activated microglial cells. In recent years, although the research of lncRNAs has achieved preliminary results, especially in tumor and neurodegenerative diseases, no lncRNA therapy or biomarker has been applied to clinical patients [37]. However, lncRNAs have been used a treatment in animals in tumor researches. It has been reported that antisense oligonucleotides targeting lncRNA-MALAT1 could reduce tumor growth and metastasis [38]. But lncRNA used as a treatment in ischemic stroke has not been reported.

Emerging studies have shown that NF-κB signaling is a pivotal driver of the inflammatory response because it can transcriptionally activate downstream pro-inflammatory cytokines such as IL-1b, TNF-α, and IL-6 [39]. A previous study provided that the NF-κB signaling pathway was over-activated in microglial cells after stroke [15]. The results of mice with p50 KO suggested that NF-κB activation aggravated ischemic neuronal damage, but its effects differed in different cells. Activation of NF-κB in microglial cells promoted ischemic neuronal death, but in neurons, activated NF-κB may increase their survival after ischemia [40]. In our research, we focused on the activation of NF-κB in microglial cells. We observed a decrease in p-IκB and an increase in p-p65 in microglial cells after OGD, and the transfer of p65 in the nucleus was also induced. In addition, overexpression of 1810034E14Rik could reverse the above changes caused by ischemia.

## Conclusion

In summary, lncRNA-1810034E14Rik contributes to brain protection from cerebral ischemic insults and to the inhibition of neuroinflammation. Furthermore, the anti-inflammatory role of 1810034E14Rik after ischemia in vivo or in vitro is highly correlated with its function in the suppression of the NF-κB pathway in microglial cells. Our results suggest that lncRNA-1810034E14Rik may become a potential treatment for the inflammatory response after ischemic stroke.

## Additional files

Additional file 1: Mice Neurological Symptom Score table. (TIF 2376 kb)

Additional file 2: All the primers used for RT-qPCR are listed in the figure. (TIF 2961 kb)

Additional file 3: The results of bioinformatics analysis and verification of lncRNAs in microglial cells after LPS treatment. **a** Biological process in which lncRNAs are involved. **b** Cellular component of lncRNAs. **c** Molecular function of lncRNAs. **d** KEGG of mRNAs related to lncRNAs. **e**, **f** Levels of lncRNAs in microglial cells challenged with LPS (100 ng/ml) for 3 h were tested by RT-qPCR. **g**, **h** Levels of lncRNAs in microglial cells challenged with LPS (100 ng/ml) for 6 h were tested by RT-qPCR. The data represents mean ± SEM. *P < 0.05, **P < 0.01, and ***P < 0.001 versus the control group. (TIF 1569 kb)

Additional file 4: The changes of 1810034E14Rik in neurons, astrocytes, and endothelial cells after OGD induce. The change of 1810034E14Rik in neurons (**a**), astrocytes (**b**), and endothelial cells (**c**) after OGD was tested by RT-qPCR. (TIF 307 kb)

Additional file 5: Verification of lncRNAs in the infarcted cortex of MCAO mice. Levels of lncRNA-U90926 (**a**), Gpr137b-ps (**b**), F630028010Rik (**c**), Mir22hg (**d**), and AW0011738 (**e**) were tested by RT-qPCR. The data represents mean ± SEM. *P < 0.05, **P < 0.01, and ***P < 0.001 versus the sham group. (TIF 678 kb)

Additional file 6: 1810034E14Rik had no effects on cerebral perfusion. A cerebral perfusion of mice during MCAO was measured by laser speckle imaging. (TIF 9361 kb)

## Abbreviations

BSA: Bovine serum albumin
LncRNA: Long non-coding RNA
LPS: Lipopolysaccharide
MCAO: Middle cerebral artery occlusion
OGD: Oxygen and glucose deprivation
ORF: Open reading frame
RT-qPCR: Real-time quantitative PCR
TTC: Triphenyl tetrazolium chloride
