# Supplementary figures and images for: LncRNA-1810034E14Rik reduces microglia activation in experimental ischemic stroke
Source: J Neuroinflammation. 2019 Apr 8;16:75. doi: 10.1186/s12974-019-1464-x (PMC6452518; doi:10.1186/s12974-019-1464-x)

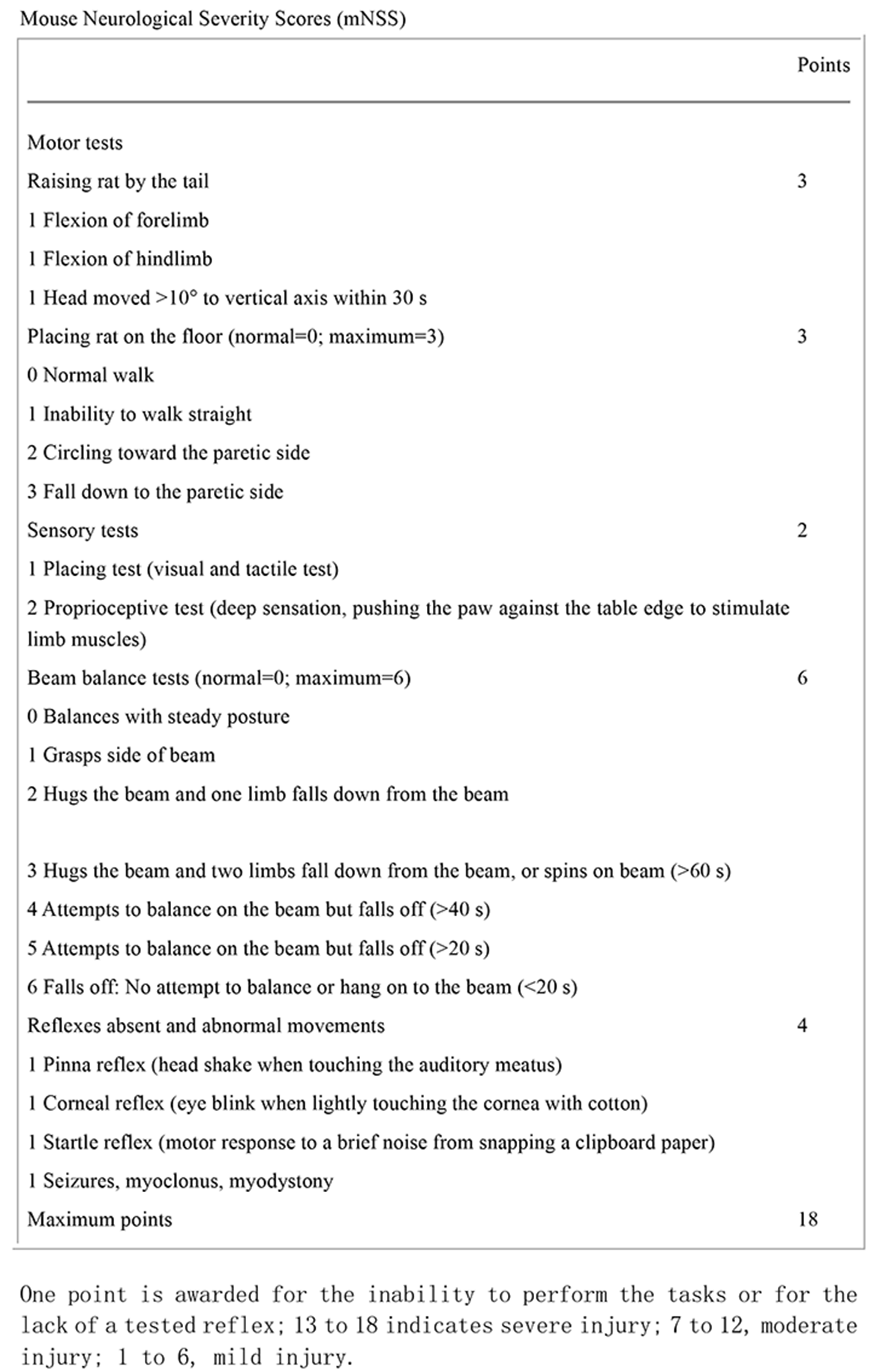

Supplement: Supplementary file 1 — Mice Neurological Symptom Score table. (TIF 2376 kb) [file 12974_2019_1464_MOESM1_ESM.tif]

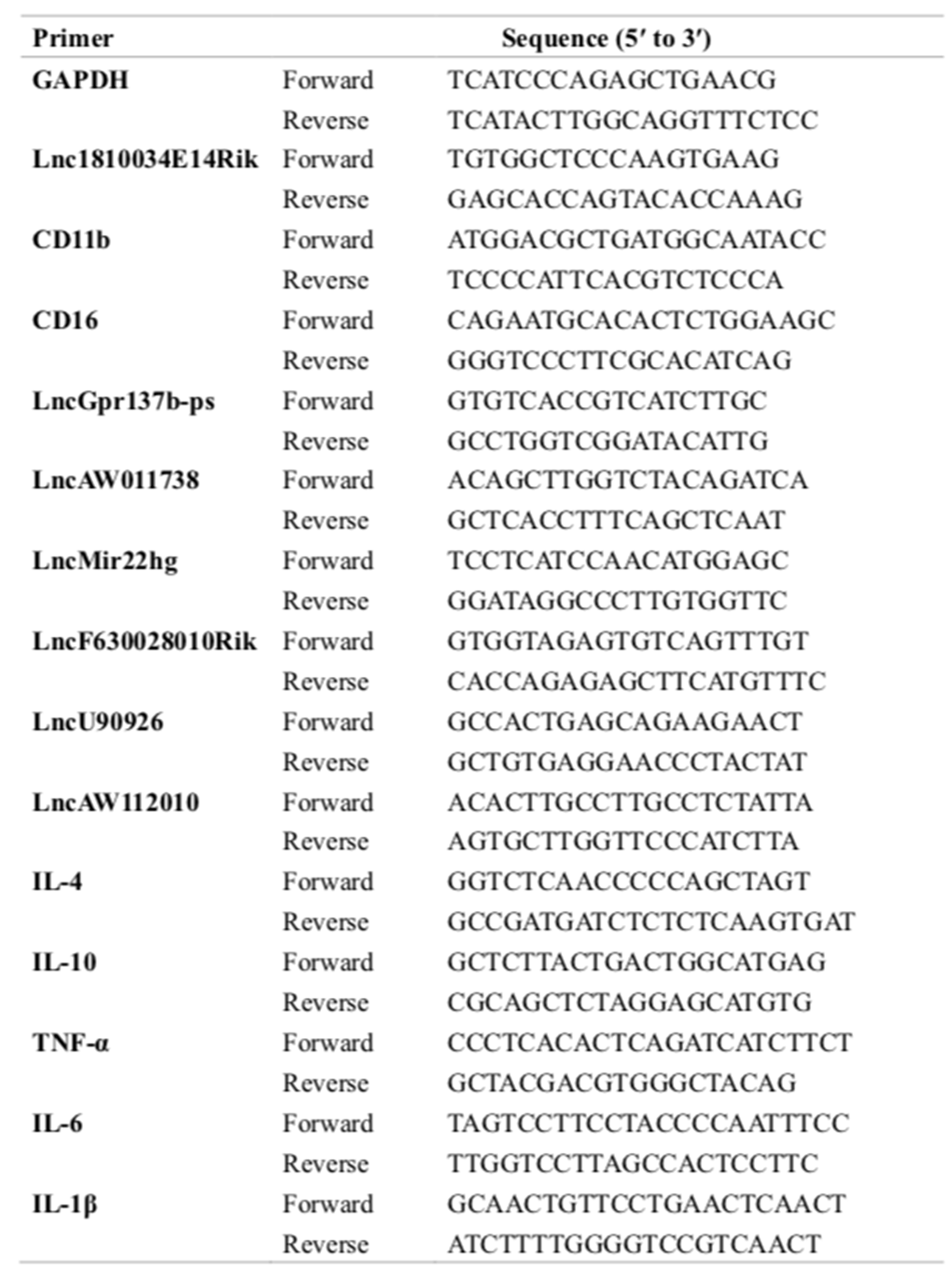

Supplement: Supplementary file 2 — All the primers used for RT-qPCR are listed in the figure. (TIF 2961 kb) [file 12974_2019_1464_MOESM2_ESM.tif]

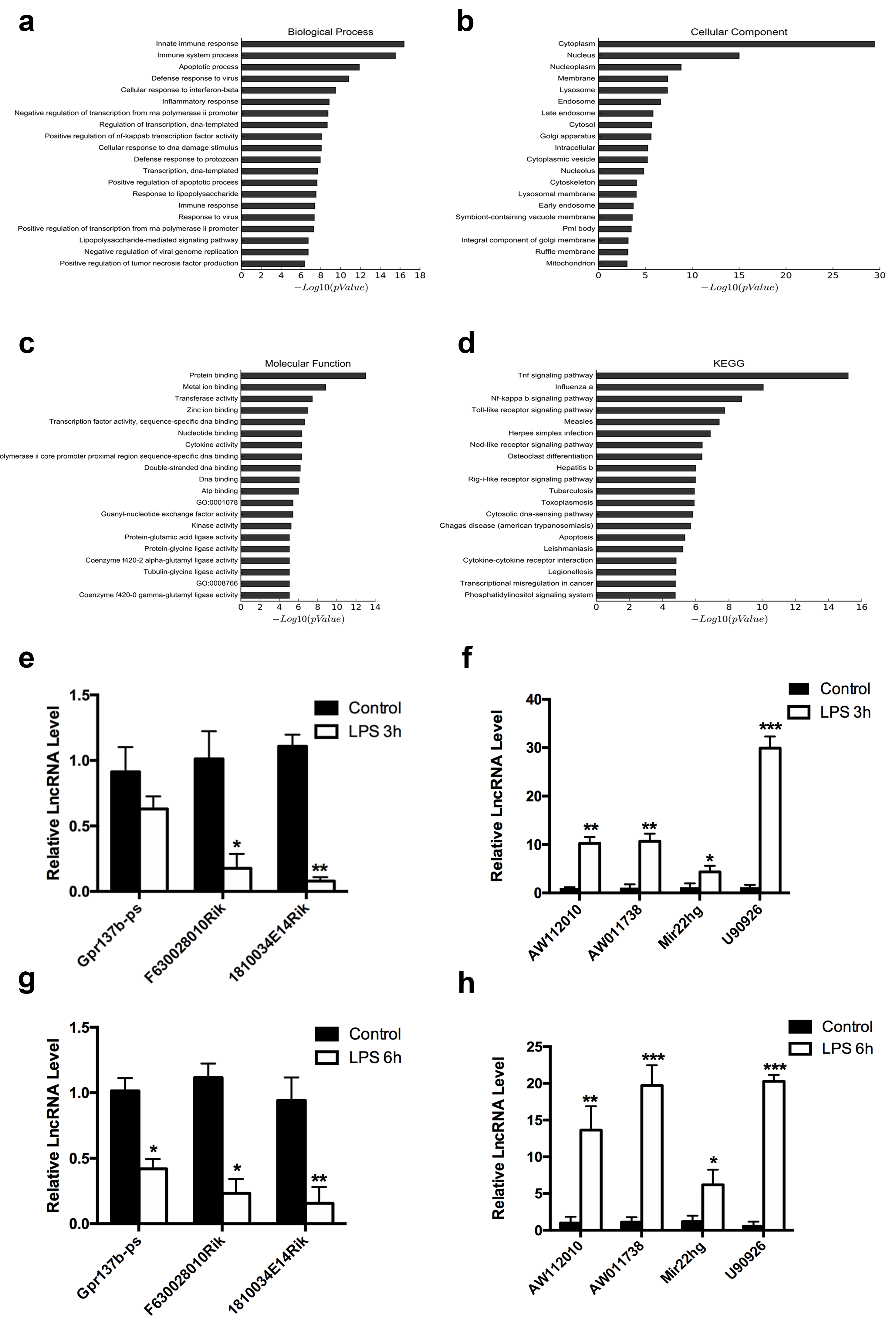

Supplement: Supplementary file 3 — The results of bioinformatics analysis and verification of lncRNAs in microglial cells after LPS treatment. a Biological process in which lncRNAs are involved. b Cellular component of lncRNAs. c Molecular function of lncRNAs. d KEGG of mRNAs related to lncRNAs. e, f Levels of lncRNAs in microglial cells challenged with LPS (100 ng/ml) for 3 h were tested by RT-qPCR. g, h Levels of lncRNAs in microglial cells challenged with LPS (100 ng/ml) for 6 h were tested by RT-qPCR. The data represents mean ± SEM. *P < 0.05, **P < 0.01, and ***P < 0.001 versus the control group. (TIF 1569 kb) [file 12974_2019_1464_MOESM3_ESM.tif]

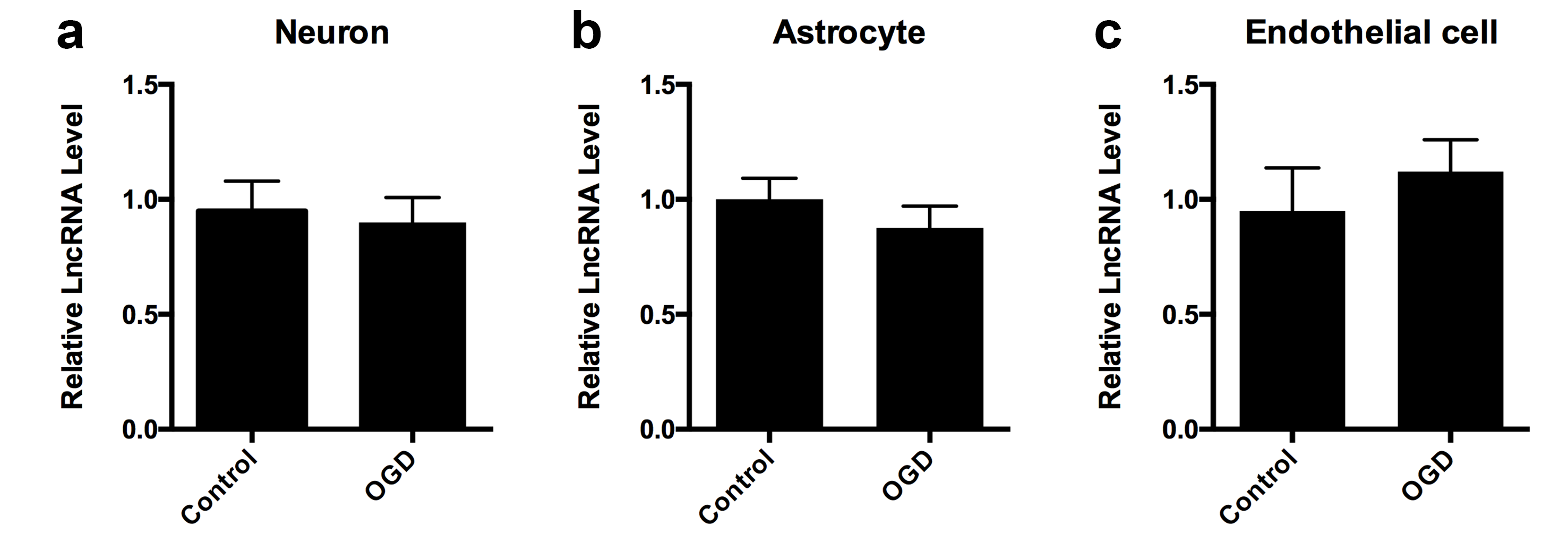

Supplement: Supplementary file 4 — The changes of 1810034E14Rik in neurons, astrocytes, and endothelial cells after OGD induce. The change of 1810034E14Rik in neurons (a), astrocytes (b), and endothelial cells (c) after OGD was tested by RT-qPCR. (TIF 307 kb) [file 12974_2019_1464_MOESM4_ESM.tif]

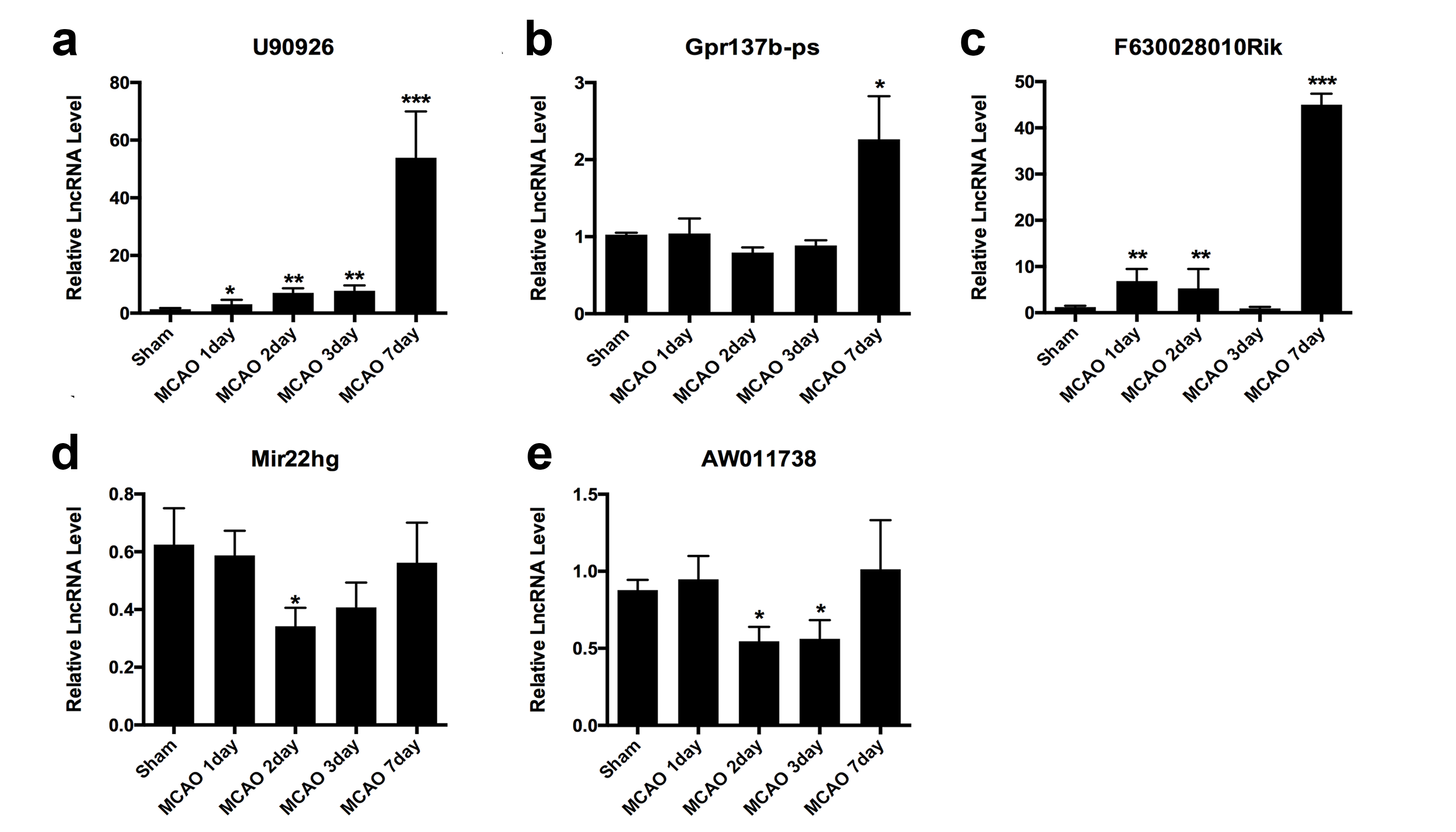

Supplement: Supplementary file 5 — Verification of lncRNAs in the infarcted cortex of MCAO mice. Levels of lncRNA-U90926 (a), Gpr137b-ps (b), F630028010Rik (c), Mir22hg (d), and AW0011738 (e) were tested by RT-qPCR. The data represents mean ± SEM. *P < 0.05, **P < 0.01, and ***P < 0.001 versus the sham group. (TIF 678 kb) [file 12974_2019_1464_MOESM5_ESM.tif]

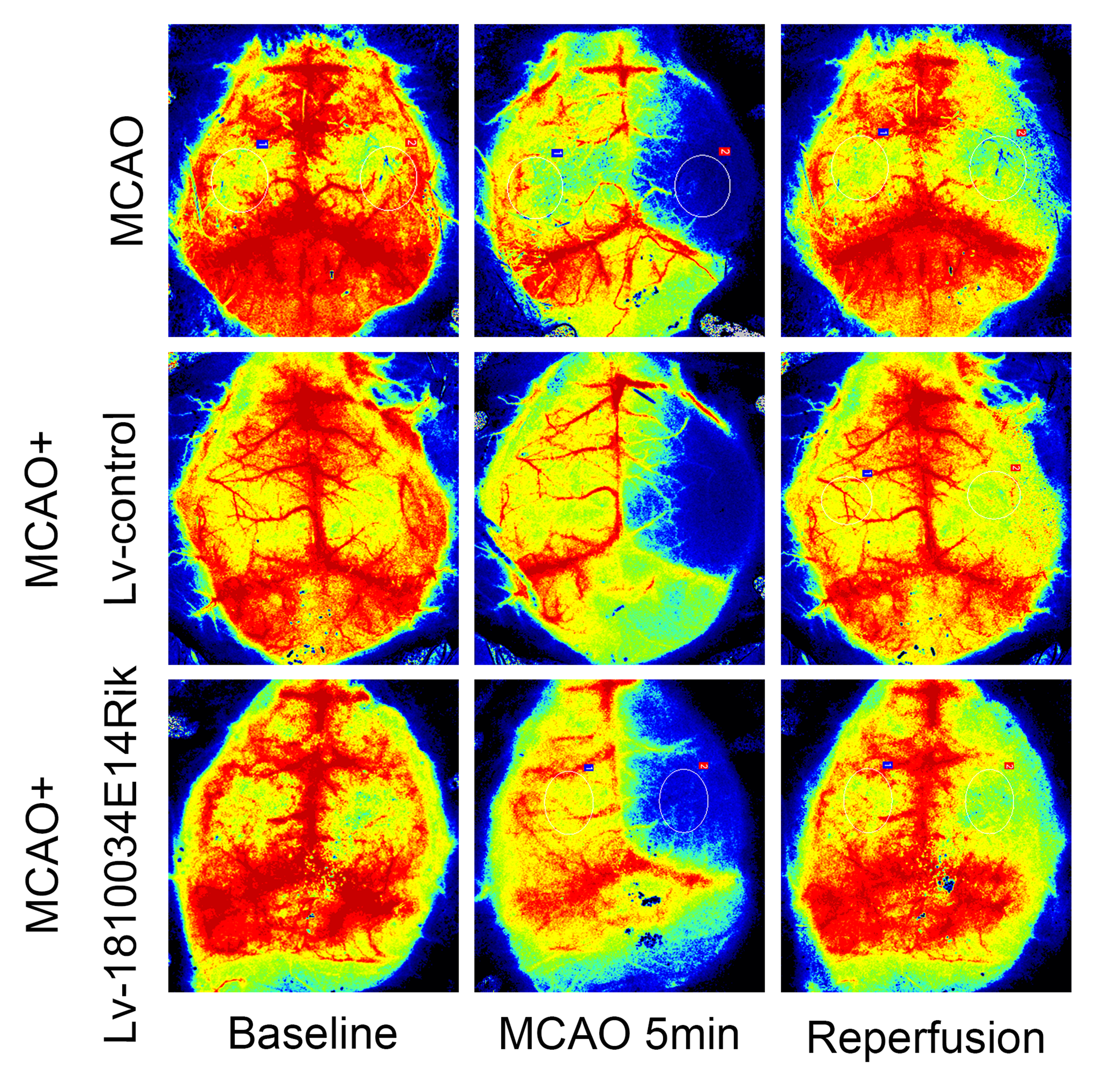

Supplement: Supplementary file 6 — 1810034E14Rik had no effects on cerebral perfusion. A cerebral perfusion of mice during MCAO was measured by laser speckle imaging. (TIF 9361 kb) [file 12974_2019_1464_MOESM6_ESM.tif]
